# Regulation of energy metabolism by inflammation: A feedback response in obesity and calorie restriction

**DOI:** 10.18632/aging.100155

**Published:** 2010-06-06

**Authors:** Jianping Ye, Jeffrey N. Keller

**Affiliations:** Pennington Biomedical Research Center, Louisiana State University System, LA 70808, USA

**Keywords:** Inflammation, caloric restriction, obesity, energy expenditure

## Abstract

Caloric restriction (CR), in the absence of malnutrition, delays aging and prevents aging-related diseases through multiple mechanisms. A reduction in chronic inflammation is widely observed in experimental models of caloric restriction. The low inflammation status may contribute to the reduced incidence of osteoporosis, Alzheimer's disease, cardiovascular diseases and cancer in the aging subjects. The association of caloric restriction with low inflammation suggests a role of energy accumulation in the origin of the chronic inflammation. This point is enforced by recent advances in obesity research. Abundant literature on obesity suggests that chronic inflammation is a consequence of energy accumulation in the body. The emerging evidence strongly supports that the inflammatory response induces energy expenditure in a feedback manner to fight against energy surplus in obesity. If this feedback system is deficient (Inflammation Resistance), energy expenditure will be reduced and energy accumulation will lead to obesity. In this perspective, we propose that an increase in inflammation in obesity promotes energy expenditure with a goal to get rid of energy surplus. A decrease in inflammation under caloric restriction contributes to energy saving. Inflammation is a mechanism for energy balance in the body. Inflammation resistance will lead to obesity. We will review the recent literature in support of the viewpoints.

## Introduction

Caloric restriction (CR) reduces the levels of multiple aspects of inflammation [1-3], suggesting a link between energy status and inflammation. This linkage is enforced by recent progress in obesity research. Chronic inflammation is widely observed in obesity (metabolic syndrome). The obesity-associated inflammation is involved in pathogenesis of type 2 diabetes, hypertension, atherosclerosis, fatty liver, cancer metastasis, and asthma in obesity. Obesity has a higher prevalence in the aging population as a result of reduced energy expenditure with less physical activity. Physical activities consume a major portion of energy in our daily life, which are usually reduced in the aging population. This reduction in energy expenditure may lead to energy accumulation in the body and consequently a gain in adiposity. In obesity, systemic chronic inflammation occurs with elevated proinflammatory cytokines (IL-6, MCP-1, CRP, PAI-1, et al.) in the circulation. The systemic inflammation is due to an inflammatory response in adipose tissues that are under quick expansion. Adipocytes produce these cytokines. In addition, macrophage infiltration into the adipose tissue contributes significantly to the cytokine production. Although we have learned a lot about the signaling pathways that link energy accumulation (adiposity) to chronic inflammation, we know little about the real biological significance of the inflammation. This article addresses this issue, and provides an overview of the interaction of inflammation and energy balance.

### 1. Chronic inflammation from energy accumulation

In obesity research, the link between chronic inflammation and energy (fat) accumulation is well established. The initial observation of TNF-α elevation in adipose tissue of obese mice provides the first evidence for the chronic inflammation in 1993 by Hotamisligil and colleagues [4]. Thereafter, the concept was enforced by abundant literature identifying increases in many other inflammatory cytokines, such as plasma C-reactive protein (CRP), interleukin 6 (IL-6), plasminogen activator inhibitor-1 (PAI-1), in models of obesity. Activation of inflammatory kinases such as IKKβ (IkBα kinase beta) and JNK1 (c-Jun N-terminal kinase 1) provides additional evidence for activation of intracellular inflammatory pathways in obesity [5-6]. Obesity-associated inflammation is chronic, systemic, low-grade, and not linked to any infection. In contrast to inflammation induced by bacteria or virus infection where neutrophil granulocytes are elevated in the circulation, neutrophil granulocytes are not increased in blood in obesity. The inflammation is systemic since the inflammatory cytokines are increased in the circulation. The inflammation is at a low grade in obesity since there is no fever and malaise, which are often observed for inflammation associated with bacteria/viral infection.

### 2. Inflammation origin: Energy accumulation may induce inflammation through metabolites of fatty acids and glucose (Figure 1)

**Figure 1.**
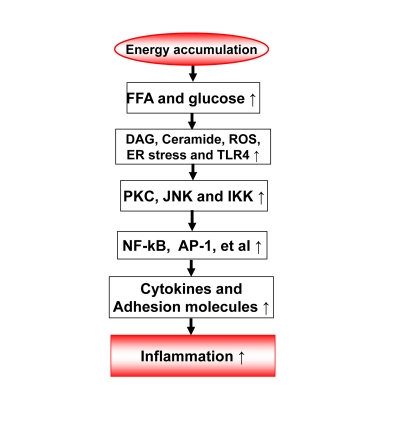
Energy accumulation induces inflammation. Energy accumulation leads to elevation in glucose and fatty acids. These substrates lead to production of diaglycerids (DAG), Ceramide, reactive oxygen species (ROS) and activation of toll-like receptor 4 (TLR4) in cells including macrophages and endothelial cells. All of these events may activate the inflammatory signaling pathways, such as IKK/NF-kB and JNK/AP-1. As a consequence, expression of inflammatory cytokines and adhesion molecules may increase for chronic local inflammation. When inflammatory cytokines are elevated in the circulation, the energy accumulation causes systemic chronic inflammation, which is observed in obesity. This kind of chronic inflammation is limited or prevented by calorie restriction

The metabolites of fatty acids and glucose include diaglyceride (DAG), Ceramide, and reactive oxygen species (Figure 1). They activate inflammatory response through several approaches. They may direct interact with signaling kinases (PKCs, JNKs and IKKs) in cells [7]. They may also act through cell membrane receptors for lipids, such as TLR4, CD36 or GPR [8-11]. The reactive oxygen species (ROS) are generated from fat or glucose oxidation in mitochondria. ROS may induce activation of the inflammatory kinases (JNK and IKK). The lipids also induce endoplasmic reticulum (ER) stress for activation of JNK and IKK [12-13]. In CR, these metabolites of glucose and fatty acids are reduced from less calorie intake. The risk of inflammation is reduced.

In obesity, adipose tissue is a major source of chronic inflammation [14-15]. In adipose tissue, adipocytes and adipose tissue macrophages (ATM) are the major cell types responsible for the production of inflammatory cytokines. The representative cytokines include TNF-α, IL-6, MCP-1 and PAI-1. Adipokines (Leptin and adiponectin) are produced by adipocytes and also involved in the regulation of inflammation. Macro-phages and adipocytes are activated during the process of adipose tissue expansion. Recent studies suggest that the adipose tissue expansion induces a local hypoxia response [16]. The hypoxia response serves as a common root for all of the stress responses in adipose tissue, such as oxidative stress, ER stress, and inflammatory stress [17-19]. Hypoxia directly promotes the chronic inflammation through activation of transcription factors (NF-kB and HIF-1) in adipocytes and macrophages [16]. The hypoxia response is a result of tissue expansion. In CR, adipose tissue expansion is reduced or under controlled. The risk factors for inflammation, such as adipose tissue hypoxia, lipid accumulation, ER stress and oxidative stress are all reduced or absent. These may explain why CR reduces the risk for chronic inflammation in the body.

### 3. Inflammation feedback to energy accumulation

**Figure 2.**
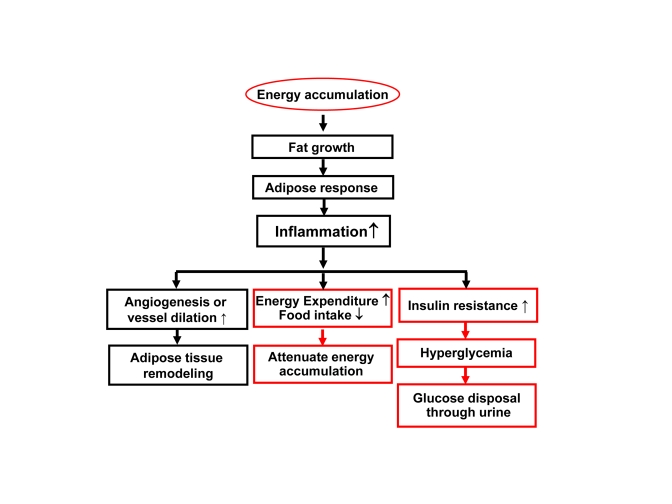
Inflammation in obesity. Rapid growth of adipose tissue leads to quick expansion of adipose tissue. When angiogenesis or vessel dilation can not meet the demand for blood supply, there will be an adipose tissue hypoxia (ATH) from lack of blood supply. ATH will induce angiogenesis and trigger inflammation. Inflammation will promote angiogenesis and vasodilation locally in the tissue for extracellular remodeling. When inflammatory cytokines and fatty acids are elevated in the circulation, they will promote energy expenditure systemically. The inflammatory response may also induce hyperglycemia and energy disposal through glucose excretion in urine. In this way, inflammation acts through insulin resistance and hyperglycemia.

The inflammation observed in adipose tissue likely serves as a feedback signal locally in adipose tissue and systemically for energy expenditure (Figure 2). In adipose tissue, inflammation inhibits adipocyte expansion and adipocyte differentiation, changes adipocyte endocrine and induces extracellular matrix remodeling [20]. The local response is translated into a systemic response through cytokines and free acids released from adipose tissue.

(a) Adipocyte inhibition. A major function of adipocytes is to store fat. In addition, the adipocytes secrete many cytokines/hormones in its endocrine activity. Inflammatory cytokines inhibit adipocyte function in multiple aspects. These include inhibition of preadipocyte differentiation, induction of lipolysis and suppression of adiponectin expression in mature adipocytes. These inhibitory activities are well documented for TNF-α and IL-1 [21-23]. At the molecular level, inflammation inhibits insulin signaling pathway [24-26] and PPARγ activities in adipocytes [27]. These effects contribute to suppression of tissue expansion, and alteration in cytokine profile. The disorders in lipid metabolism and cytokine balance contribute to the whole body insulin resistance, a result of impaired insulin signaling in multiple organs (skeletal muscle, liver, and adipose tissue) [28-30]. Insulin resistance may induce hyperglycemia, which in turn leads to glucose excretion through urine (type 2 diabetes). The type 2 diabetes is an extreme condition in the body to get ride of energy surplus in an effort to prevent energy accumulation in the body.

(b) Adipose tissue remodeling: Macrophage infiltration is a major marker of local inflammation in the adipose tissue in obesity. Adipose tissue macrophages (ATM) have been under active investigation since 2004 when macrophage infiltration was initially identified in obese mice [31-34]. The discovery provides a source for TNF-α in adipose tissue since mature adipocytes produces very little TNF-α [31-34]. The biological significance of macrophage infiltration remains to be elucidated. However, more and more evidence suggests that macrophages are required for adipose tissue remodeling and adipogenesis of preadipocytes. Macrophages may serve as a signal amplifier in the adipose tissue for stimulation of angiogenesis [35]. Macrophages produce many angiogenic factors, such as PDGF, TGF-β and HGF, which are increased in adipose tissue in obese individuals [36-37]. Interestingly, this activity of macrophages is required for adipose tissue growth in lean mice [38-39] and obese mice [35]. Macrophages may also regulate blood flow through production of vasodilators (such as NO). Macrophages may clean the cell debris of dead adipocytes within the adipose tissue [40]. An increase in adipocyte death was reported in the adipose tissue of obese mice, and the dead cells were surrounded by ATMs to form the "Crown" like structure [40-41]. The cell death in adipose tissue may be a result of the hypoxia response [42]. In CR, the adipose tissue expansion is under control, there are not such risk factors for macrophage activation in adipose tissue.

(c) Fuel mobilization. Inflammation regulates fuel mobilization. Fuel (fatty acids) mobilization from adipose tissue to other tissues is controlled by the nervous system and hormones/cytokines. The role of inflammatory cytokines has drawn a lot of attention in the fuel mobilization. Cytokines such as TNF-α, IL-1, IL-6, et al., activate fuel efflux in adipocytes through lipolysis, in which free fatty acids (FFAs) are generated from triglycerides under hydrolysis and released into blood stream. FFAs are normally oxidized in mitochondria for ATP production. An increase in FFA supply may lead to acceleration of energy expenditure. However, when FFA supply overrides the consumption, they deposit in non-adipocytes in the form of ectopic fat deposition. The ectopic fat contributes to pathogenesis of fatty liver disease and atherosclosis (deposit on the blood vessel wall). In the physiological conditions, IL-6 secreted by contracting muscle is involved in coordination of fuel mobilization between adipose tissue and skeletal muscle during exercise [43-44]. In CR, the fatty acid supply is limited as a result of reduced calorie intake, the risk for ectopic fat deposition will be reduced. This may help in prevention of fatty liver and atherosclosis.

(d) Energy intake. Inflammatory cytokines are involved in the regulation of energy intake and expenditure. IL-1 and IL-6 reduces food intake and prevent hyperphagia [45-46]. Cytokines (IL-1, IL-6 and TNF-α) also induce energy expenditure [46-50]. These activities of cytokines are dependent on their actions in the central nervous system [46-47,51-52]. Therefore, inflammatory cytokines may serve as an anti-obesity signal by modifying both energy intake and energy expenditure. Additionally, these data indicate that the inflammatory cytokines may serve as a link between peripheral tissues and central nervous system in the control of energy balance.

### 4. Energy expenditure by inflammation

The activities of inflammatory cytokines on adipocytes and neurons suggest that inflammation may inhibit energy accumulation. They induce energy expenditure and inhibits food intake. These possibilities are strongly supported by phenotypes of transgenic mice with chronic inflammation and by cytokine infusion studies. Transgenic mice of IKK2/NF-kB have provided new evidence.

The IKK2/NF-kB pathway is a dominant inflammation signaling pathway. The pathway has been under active investigation in the obesity field after IKKβ was found to induce insulin resistance in obese mice [5]. The serine kinase IKK has three major isoforms including IKKα (IKK1), IKKβ (IKK2) and IKKγ, in which IKKβ is required for NF-kB activation [53]. In obesity, IKKβ is activated by several intracellular signals, such as ROS, ER stress, DAG, and Ceramide. IKKβ is also activated by the extracellular stimuli including TNF-α, IL-1, and fatty acids [8], and hypoxia [54]. IKKβ induces NF-kB activation by phosphorylation of the Inhibitor Kappa B alpha (IkBα) [55].

NF-kB (nuclear factor kappa B) is a ubiquitous transcription factor that is formed by two subunits of Rel family, which include seven members, p65 (RelA), p50 (NF-kB1), c-Rel, RelB, p100, p105, p52 [56]. These members form a homodimer or heterodimer in the regulation of gene transcription. In most case, NF-kB is a heterodimer of p65 and p50. P65 contains the transactivation domain and mediates the transcriptional activity of NF-kB. P50 usually inhibits the transcriptional activity of p65 [57], and the inhibition disappears in the NF-kB p50 knockout mice [58]. In the classical pathway, NF-kB activation is mediated by IKKβ-induced phosphorylation, proteasome-mediated degradation of IkBα [53]. In response to stress responses, NF-kB promotes lipid mobilization through suppression of PPARγ activity in the nucleus [59]. It also induces transcription of inflammatory cytokines (TNF-α, IL-1, IL-6, MCP-1, et al.). In the alternative pathway, NF-kB is activated by hypoxia in the absence of IkBα degradation. This type of NF-kB activation in adipocytes and macrophages contributes to chronic inflammation in the adipose tissue of obese individuals [16].

NF-kB activity may promote energy expenditure. This activity of NF-kB is supported by documents on energy expenditure in cachexia [60-61] and infection. However, the role of NF-kB in energy expenditure was not tested in transgenic models. To this point, we investigated energy metabolism in transgenic mice with elevated NF-kB activities. The transcriptional activity of NF-kB is enhanced either by over-expression of NF-kB p65 (RelA) in the fat tissue, or inactivation of NF-kB p50 (NF-kB1) by global gene knockout [65]. In these two models, inflammatory cytokines (TNF-α and IL-6) were elevated in blood and energy expenditure was increased in day and night [65]. The oxygen consumption and CO2 production were both increased in the mice. Locomotion was not altered, but food intake was increased in the mice. Expression of inflammatory cytokines (TNF-α and IL-6) was elevated in adipose tissue and macrophages. On a high fat diet (HFD), both lines of transgenic mice were protected from obesity and insulin resistance [65-66]. The data suggests that the transcription factor NF-kB promotes energy expenditure and inhibits energy accumulation. The inflammatory cytokines may mediate the NF-kB activity in energy expenditure. In the mice, lipid accumulation is prevented by the enhanced energy expenditure. The studies suggest that inflammation may prevent insulin resistance by eliminating lipid accumulation. IKKβ was investigated in the control of insulin sensitivity [5,62-63] and food intake in transgenic mice [64]. However, IKKβ was not investigated in the control of energy expenditure in these studies.

NF-kB may promote energy expenditure through the inflammatory cytokines. In the two transgenic models, systemic inflammation was observed with elevated proteins for TNF-α and IL-6 in the serum [65-66]. Expression of TNF-α and IL-1 mRNA was increased in adipose tissue and macrophages. These cytokines are positively associated with energy expenditure in the body [61]. In transgenic mice with deficiency in these cytokines or their receptors, energy accumulation is enhanced, suggesting a reduction in energy expenditure. This positive energy balance was reported in transgenic mice with deficiency in TNF-α [50], IL-1 [45] or IL-6 [46]. On the other side, when these cytokine activities are enhanced in transgenic mice, energy accumulation is decreased leading to a lean phenotype [48-49,67-68]. The cytokines may act in the hypothalamus of central nervous system to regulate the energy balance [46-47,51-52]. In addition to the central mechanism, activation of mitochondria by the cytokines in the peripheral tissues may also contribute to the energy expenditure. TNF-α and IL-1 enhances mitochondrial function through phosphorylation-mediated activation of PGC-1α [69]. This activity of inflammatory cytokines may contribute to energy consumption in mitochondria-enriched tissues/organs such as liver, skeletal muscle and brown fat. Inflammation may be a drug target in the management of energy metabolism [70-71].

### 5. CR and chronic inflammation

Studies have demonstrated that CR decreases the circulating levels of inflammatory cytokines and inflammatory signaling activities in a wide variety of tissues [1-3]. CR is able to decrease global levels of inflammatory responses in the body. Interestingly, the beneficial effects of CR may be related to a decrease in visceral fat and adipose reactivity [3,72]. It has been documented that adiposity during aging contributes to a number of morbidity factors including insulin resistance, dyslipidemia, atherosclerosis, hypercoagula-bility and hypertension [73-74]. However, it is important to remember that the most inflammation data are derived from the visceral fat and ectopic fat [72-74].  For example, subcutaneous fat has been observed to have beneficial effects on lipid and energy homeostasis, and even counteract the negative effects of visceral adipose tissue [75].  It is important to note that CR has beneficial effects in non-obese humans as well as non-obese rodents [76-77], indicating that decreased adiposity may not be the only mediator of beneficial effects of CR. This fact suggests that a decrease in energy accumulation is more important in the control of inflammation since this may apply to both obese and non-obese conditions.

## Summary

Energy accumulation induces chronic inflammation. This view is supported by data from many model systems of CR and obesity. Inflammation may promote energy expenditure in a regulatory-feedback manner to fight against energy surplus (Figure 2). This concept extends our understanding of biological significance of inflammation in obesity. It also helps us to understand why CR reduces inflammation. The inflammation may act in the peripheral organs/tissues as well as in the central nervous system to regulate energy balance. In the peripheral, inflammation induces fat mobilization and oxidation to promote energy expenditure. Inflammation may induce energy disposal through glucose excretion in urine as a result of insulin resistance and hyperglycemia. In the central, inflammation may inhibit food intake and activate neurons for energy expenditure. If this feedback system is deficient, energy expenditure will be interrupted and fat will be accumulated in the body for adiposity. We call this condition of "Inflammation Resistance". In CR, the energy accumulation is prevented. In turn, the risk factors for the chronic inflammation are limited. In our view, the low inflammation serves as a mechanism for energy saving in CR.
